# Sudden psychotic episode probably due to meningoencephalitis and *Chlamydia pneumoniae *acute infection

**DOI:** 10.1186/1745-0179-1-15

**Published:** 2005-09-15

**Authors:** Miguel Xavier, Bernardo Correa, Marta Coromina, Nuno Canas, João Guimarães

**Affiliations:** 1 Depart. Psychiatry and Mental Health, Faculty Medical Sciences – UNL Calçada da Tapada, 155, 1300-Lisbon, Portugal; 2 Depart. Psychiatry – Hospital S. Francisco Xavier, 1400-Lisbon, Portugal; 3 Depart. Neurology, Hospital Egas Moniz, 1400-Lisbon, Portugal

## Abstract

**Background:**

Since 9% to 20% of all cases of acute psychosis presenting to an Emergency Department (ED) are due to a general medical condition, cautious medical workup should be mandatory in such patients. Differential diagnosis must consider conditions as diverse as renal failure or CNS infection.

Acute Chlamydia pneumoniae infection usually causes a self-limited respiratory syndrome. Rarely, acute neurological complications occur, with acute meningoencephalitis most frequently reported. Diagnosis requires a high level of suspicion and is difficult to confirm.

**Case report:**

We describe a 22 year-old female Caucasian who, three days after a mild pharingitis, developed an acute psychosis with exuberant symptoms interspersed with periods of lucidity, in a background of normal consciousness and orientation.

Initial medical and imagiological workup were inconclusive. After 20 days of unsuccessful treatment with antipsychotics she developed a high fever and was re-evaluated medically. Lumbar puncture revealed an inflammatory cerebrospinal fluid. MRI showed irregular thickening and nodularity of the lateral ventricles' lining. An anti-Chlamydia pneumoniae IgM antibody titter of 85 IU/ml was detected. All symptoms cleared after treatment with antibiotics and corticosteroids.

**Conclusion:**

This is, to our knowledge, the first reported case of acute CP-associated meningoencephalitis manifesting as an acute psychotic episode. It illustrates the principle that non-organic psychiatric syndromes must remain a diagnosis of exclusion in first-time acute psychosis.

## Background

It is estimated that between 9% and 20% of all cases of acute psychosis presenting to an Emergency Department (ED) are due to a general medical condition [1-3]. This means that all patients coming to the ED with signs and symptoms of acute psychosis must undergo a careful diagnostic workup. Apart from non-organic psychiatric syndromes themselves, the differential diagnosis must include physical trauma, drugs and toxins, organ failure (e.g., renal failure), structural lesions like intracranial hematomas or neoplasms, infections and nutritional deficiencies like vitamin B12 deficiency or pellagra [1-4]. The psychiatrist plays a fundamental role in this process and is often the last resort that keeps the patient from sliding down the almost no-return path of an erroneous non-organic psychiatric diagnosis [2,4,5]. The sudden onset of psychosis in a 40 year-old patient with no personal or family history of psychiatric disease who shows recent memory loss, altered vital signs and a clouded consciousness with disorientation and visual hallucinations will probably be easily identified as a possible medical condition manifesting through behavioural changes [5]. Many cases, however, do not present such clear-cut features and false beliefs (i.e. delusions) may actually constitute the sole manifestation of central nervous system disfunction (CNS) [6]. Such patients often come to constitute a true challenge to the attending psychiatrist.

*Chlamydia pneumoniae *(CP) is an intracellular organism with worldwide distribution and is pathogenic both to humans and other vertebrates. The metabolically inactive but infectious extracellular form (elementary body) differentiates into reticulate body after endocytosis by macrophages/monocytes, endothelial cells or vascular smooth muscle cells. Within these cells it then replicates by binary fission [7]. It is currently speculated that the respiratory tract be the site of entry for CP, from whence it is then transported by monocytes or macrophages to other sites in the organism. It remains unclear how CP infection leads to disease, as it does not produce toxins and has only weak lipopolysaccharide activity [8]. In humans CP most frequently causes a self-limited and uneventful acute respiratory tract syndrome. More rarely it can cause multiorganic disease with an occasionally fatal outcome [8,7,9]. Over the last few years CP has become the object of intense debate among neurologists as being possibly involved in the aetiology of such diverse conditions as Alzheimer's disease, multiple sclerosis or atherosclerosis (this last possibility earning it the ironical nickname "cardiology's Helicobacter") [10-18]. Acute neurological complications of CP infection seem to be rare [10]; as to our knowledge no more than twenty putative cases with variable manifestations have been published so far [19-25]. Meningoencephalitis has been by far the most frequently reported situation, with isolated reports of meningoradiculitis, polyradiculoencephalitis, cerebelar disfunction, Guillain-Barré syndrome and acute disseminated encephalomyelitis. In most such cases direct evidence of central nervous system (CNS) involvement by CP in has been difficult to obtain; to our knowledge only three cases have been reported where intrathecal production of specific anti-CP antibodies was demonstrated [19-21]. In a further case the authors claim to have detected CP antigens in their patient's CSF and more recently a case was reported where intrathecal presence of CP DNA was detected by PCR [23,25].

## Case report

A 22 year-old single, Caucasian female Business-school student developed a low fever, headache and sore throat, which remitted spontaneously after three days. Seven days after the start of these symptoms she suddenly became agitated, physically and verbally aggressive, with total insomnia and disrupted behaviour. She was taken by her parents to the Emergency Department of S. Francisco Xavier Hospital, Lisbon, and was compulsorily hospitalised in the acute psychiatric ward under the Portuguese Mental Health Law. The mental state examination revealed a vigil and fully oriented patient. She appeared distrustful, collaborated poorly and was easily distracted. She had false delusional memories and paranoid delusional, poorly structured ideas of persecutory, magical and grandiose content, as well as a disrupted awareness of the boundaries and vitality of the Self. She also claimed, in what appeared to be a delusional misidentification of the Capgras syndrome type, that robots had replaced her parents. She appeared to be suffering complex auditive hallucinations in the 2nd and 3rd person, with a menacing content. Her mood was dysphoric, with poorly contained, labile affects. Her past medical record and family history were irrelevant. Neurological and general physical examinations were normal. She was started on haloperidol 10 mg tid ev (she refused oral medication), with no improvement of her mental state, although she did have unpredictable, short-lived intervals of remarkable lucidity with almost total absence of psychiatric symptoms apart from a depressed mood. 20 days after admission she developed a high fever with a white-cell count of 27 × 109/L, an erythrocyte sedimentation rate (ESR) of 100 mm/h and a C-reactive protein (CRP) of 15.3 mg/L. Routine biochemistry studies were normal. The cerebrospinal fluid (CSF) was clear, with normal protein and glucose concentrations and 20 × 106 lymphocytes per litre. Microbiological investigation of CSF was negative. Her chest film and brain CT-scan showed no changes. Brain MRI showed irregular thickening and nodularity of the lateral ventricles' lining. A complete neuropsychological evaluation was performed revealing a mild impairment of her external visual attention and visuo-constructive reproduction capacity. The EEG displayed symmetrical, irregular, high amplitude, slow wave activity with frontotemporal predominance. An ophthalmologic examination revealed lymphocytes in her vitreum. A skin biopsy was taken for Lupus Band Test, which was negative. Serologic testing of serum and CSF was performed for systemic lupus erythematosus (including anti-ribosomal P antibodies) and other connectivites as well as for most known neurotropic viruses and bacteria. An IgG antibody titer to CP of 22 IU/ml was detected in the serum, with an IgM antibody titer of 85 IU/ml. No anti-CP antibodies were detected in the CSF. All her psychotropic medication was stopped and she was started on levofloxacin (due to dysuria and leukocyturia) and acyclovir. Five days levofloxacin was changed to gentamicin plus piperacillin/tazobactam for due to Escherichia coli-positive blood-cultures (serological results were not yet available at that time). She finally received three consecutive ev pulses of methylprednisolone (1 g each), before being transferred to a Neurology unit. During the following week she progressively became apyretic and her psychotic symptoms gradually remitted. At the time of discharge, eight weeks after admission, she still had an ESR of 55 mm/h and a CRP of 9.03 mg/L, with normal blood count and biochemical parameters as well as a normal CSF. Brain MRI nine weeks after admission showed complete remission of the initial changes. At the time of discharge, the EEG still displayed symmetrical, irregular, high amplitude, slow wave activity with frontotemporal predominance, although much less severe than in the first EEG record.

Psychiatrically she remained slightly disinhibited, both verbally and affectively, with pedolalia and mildly childish behaviour. There were no signs of delusional ideas or hallucinations and she could only vaguely remember the period when she had been psychotic. Two months after discharge she had resumed her studies and concluded her Business School degree.

## Discussion

This is, to our knowledge, the first reported case of an acute CP-associated meningoencephalitis manifesting as a first-episode acute psychosis.

In our patient the initial manifestations were interpreted as a purely non-organic psychiatric syndrome, which led to a delay of several weeks not only in the definitive diagnosis but also in the appropriate treatment. The patient's young age, her clear consciousness and normal orientation, the presence of archetypical first-rank symptoms of schizophrenia and specially her normal physical and neurological examinations were all factors contributing to the premature exclusion of a medical condition as an explanation for the patient's behavioural changes. This illustrates how easy it can be to miss an organic brain syndrome at an early stage once a psychiatric diagnosis is taken into consideration and in the absence of such gross indicators as age greater than 40 years, disorientation with clouded consciousness or abnormal vital signs. Retrospectively we can identify several subtle cues in our patient pointing to a medical aetiology, namely the sudden onset of severe and exuberant psychiatric changes, the patient's high level of functioning prior to the episode and negative family history for psychiatric disorders, her intense emotional unsteadiness, the temporal relationship to a minor infectious episode, the existence of occasional "islands" of lucidity and the symptoms' minimal response to antipsychotic agents. All of these have been consistently reported in the literature as discrete but highly significant indicators of acute organic brain disorder [1,2,4,5,26,27].

A pertinent question in this context is which diagnostic methods should be included in the initial workup of first-episode acute psychosis. Although most would probably include a no-contrast brain CT scan in their initial evaluation, this is actually a method lacking in sensitivity [1-3,27]. On the other hand, MRI scanning is probably unavailable in most EDs. Also, the decision threshold for performing a lumbar puncture should be much lower, since it remains the most reliable method of detecting inflammatory changes of the CNS [1,3].

Our case is also worthy of discussion from the perspective of infectiology, especially insofar as the final bacteriological diagnosis is concerned. CP infection of the CNS is difficult to diagnose and requires a high level of suspicion. Detection of the organism's DNA in the CSF using polymerase chain reaction (PCR) probably constitutes the most reliable method of confirming the diagnosis [25,28,29]. Unfortunately, this is a method that is not always available, as it is the case in our hospital. However, the elevated titer of CP IgM antibodies in the serum and the absence of any other possible aetiology after exhaustive investigation make CP the almost certain cause of meningoencephalitis in our patient. To our knowledge only three cases where intrathecal synthesis of CP antibodies was demonstrated have been published so far and most reports of the rare acute neurological complications of CP infection have been based on serum antibody titers only [19-21]. Moreover, Sočan et al, using a direct-immunfluorescence test with CP-specific monoclonal antibodies, reported a case where they detected CP antigens in their patient's CSF, which showed negative antibody titers by microimmunofluorescence assay [23].

Doubt has been cast by several authors on the reliability of single antibody titer measurements in the serum for the diagnosis of acute CP infection and it now seems unanimous that this method's sensitivity is unsatisfactory [28,29]. The case for specificity, however, remains open to controversy. Although Gaydos *et al *detected antibody titles considered to be diagnostic of acute infection with CP in 18,8% of 80 PCR and culture negative individuals, it must be stressed that this was a sample of asymptomatic persons and so their results can in no way be extrapolated to severely ill patients presenting with clinical manifestations compatible with CP infection and in whom most alternative infectious agents have been exhaustively excluded. On the other hand, Sočan et al, in their reply to the letter to their editor by Weiss et al, claim to have found no single positive anti-CP IgM titer in samples from 100 blood donors [28]. Finally, it is probably also relevant that the abovementioned works have used microimmunofluorescence assays for measuring antibody titers in the serum, while our patient was studied using an enzyme-linked imunosorbent assay. The whole controversy around which is the most reliable method for detecting CP infection of the CNS actually developed in connection with attempts to link the organism to the aetiology of Multiple sclerosis, Alzheimer's disease or atherosclerosis, leading to the active search for CP in patients with no other evidence of infection by CP than the above-mentioned conditions themselves [11-13,15-17,30]. To be conclusive, this kind of investigation must strive for a degree of certainty and unambiguity in their methods whose sophistication and complexity are probably inadequate for everyday use in a clinical context.

Another important question in our case is what caused the patient to improve. The fact that she only received levofloxacin for five days (serological results were only available after the patient had started to improve) and her rapid recovery after corticosteroid therapy suggests that the acute neurological complications of CP infection might be attributed to an autoimmune mechanism, rather than to the organism's direct action. Frydén et al have previously reported improvement after corticosteroid therapy in CP-associated meningoencephalitis (although their patient also received cloramphenicol) [31]. The CP-associated Guillain-Barré syndrome reported by Haidl also recovered only after treatment with methylprednisolone [24]. The encephalitis case reported by Airas et al suffered further clinical and imagiological progression in spite of CP-active antibiotic treatment (levofloxacin) [19]. The case reported by Michel et al eventually recovered from lumbosacral meningoradiculitis without antibiotic treatment [21]. Finally, Grayston et al described a case where acute CP reinfection led to a sarcoidosis-like clinical picture and raise the possibility of an immunopathologic reaction in their patient [9].

In spite of our current ability to detect structural and functional changes of the CNS in organic brain syndromes, it remains unclear how these changes relate to the behavioural changes and in what way such observations might be extrapolated to our understanding of the pathophysiology of non-organic psychosis. In the present case the only observable changes seemed to concentrate around the ependymal axis. Peri-aqueductal structures have recently been considered by a few authors to be the neurostructural backbone of consciousness and especially of the consciousness of the self, whose disturbance constitutes one of essential features of schizophrenic psychosis [32,33]. This concept is in full accordance to the observations by Cummings et al, who found that in organic psychosis complex symptomatology such as Schneiderian first-rank symptoms is more frequently associated with subcortical lesions. This probably means that complex psychotic syndromes require that higher CNS functions like linguistic and verbally-mediated conceptual abilities and the corresponding cortical structures be intact. Subcortically generated abnormal emotional experiences can thus be fully elaborated at higher levels into complex delusional and hallucinatory syndromes [6]. Interestingly, organic psychosis due to subcortical brain lesions also seem to be particularly resistant to antipsychotic treatment, which was one of the striking features of our patient [6].

## Conclusion

As we stressed before, this is to our best knowledge the first reported case of an acute CP-associated meningoencephalitis manifesting as a first-episode acute psychosis and illustrates the overwhelming importance of a careful medical and neurological diagnostic workup before a sudden first-time psychotic episode receives a "non-organic" psychiatric diagnosis. In such cases non-organic psychiatric diagnosis should remain a diagnosis of exclusion [1,4], and thus approached with the assumption that the cause of psychosis is an organic one [4]. As diagnostic methods grow more complex and as we become more able to diagnose unusual medical aetiologies for common psychiatric syndromes, more and more will be demanded from clinical psychiatrists in terms of general medical knowledge and ability to work fully integrated with other specialities.

## Competing interests

The author(s) declare that they have no competing interests.

## Authors' contributions

MX wrote and submitted the manuscript

BC helped to draft the manuscript and reviewed published references

MC and NC have made substantial contributions to acquisition and interpretation of clinical data:

JG revised the manuscript critically from the neurological point of view

All authors read and approved the final manuscript. Moreover, all authors were involved in the care of the patient described in this case report
